# Synthesis and characterization of molecularly imprinted polymer nanoparticles against porcine circovirus type 2 viral-like particles

**DOI:** 10.1007/s00216-024-05576-3

**Published:** 2024-10-11

**Authors:** Jutapak Klangprapan, Wisnu Arfian A. Sudjarwo, Peter A. Lieberzeit, Kiattawee Choowongkomon

**Affiliations:** 1 Genetic Engineering Interdisciplinary Program, Graduate School, Kasetsart University, 50 Ngam Wong Wan Road, Chatuchak, Bangkok, 10900 Thailand; 2 Present Address: Department of Research Affairs, Faculty of Dentistry, Chulalongkorn University, Bangkok, 10330 Thailand; 3 University of Vienna, Faculty for Chemistry, Department of Physical Chemistry, Waehringer Strasse 42, A-1090 Wien, Austria; 4 Present Address: Research Center for Polymer Technology, National Research and Innovation Agency, Republic of Indonesia (BRIN), Serpong, Tangerang Selatan, 15314 Indonesia; 5 Department of Biochemistry, Faculty of Science, Kasetsart University, 50 Ngam Wong Wan Road, Chatuchak, Bangkok, 10900 Thailand; 6 Center for Advanced Studies in Nanotechnology for Chemical, Food and Agricultural Industries, KU Institute for Advanced Studies, Kasetsart University, Bangkok, Thailand

**Keywords:** Fluorescence quenching, Molecular imprinting, Nanoparticles, Porcine circovirus type 2, QCM analysis

## Abstract

PCV2 is a significant epidemic agricultural pathogen that causes a variety of swine diseases. PCV2 infections have significant economic impact on the swine industry, making effective strategies for rapid detection of PCV2 in pigs essential. Herein, we report on the synthesis of the so-called nano-MIPs which can be utilized for molecular recognition of PCV2. The morphology and structure of nano-MIPs were characterized using scanning electron microscopy (SEM). Nano-MIPs are spherical with sizes around 120–150 nm. Binding experiments demonstrate that the fluorescence intensity of PCV2 samples decreases proportionally to increasing the concentration of nano-MIPs due to quenching, while non-imprinted polymer nanoparticles (nano-NIPs) do not affect the signal. The Stern–Volmer constant of nano-MIPs binding to PCV2 was 1.3 × 10^−3^ mL/µg, whereas nano-NIPs led to 7 × 10^−5^ mL/µg, i.e., 1.8 orders of magnitude lower. The detection limit for binding MIP particles to PCV2 by fluorescence measurements is 47 µg/mL. This affinity test allows for designing both direct and competitive quartz crystal microbalance (QCM) assays for PCV2 leading to QCM measurements. The QCM results show nano-MIPs binding to PCV2 immobilized on the sensor surface with appreciable reproducibility. QCM sensor characteristics reveal signal saturation above around 200 µg/mL at a response of − 354 Hz and an LOD of approximately 35 µg/mL. Nano-MIPs also show selectivity factors of 2–5 for CSFV and PRRSV probably because the three viruses have similar diameters around 50 nm.

**Graphical Abstract:**

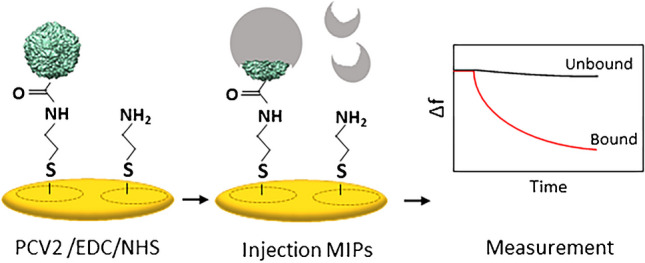

## Introduction

Porcine circovirus type 2 (PCV2) is associated with many diseases, especially postweaning multisystemic wasting syndrome (PMWS) [1], porcine respiratory disease complex (PRDC) [2], and porcine dermatitis and nephropathy syndrome (PDNS) [3]. With the development of large-scale swine industry, there is an increasing incidence of diseases caused by PCV2, which has brought huge economic loss to the swine industry around the world. PCV2 is a small, non-enveloped virus comprising a circular single-strand DNA genome of about 1.76 kb. This genome contains two functional open reading frames (ORFs). ORF1 encodes several forms of Rep protein and is involved in viral replication. It plays an important role in encoding the capsid protein (28 kDa) [4, 5] which is a unique structural protein of each virus. The capsid protein contains specific immunodominant epitopes. It has revealed strong reactivity with monoclonal antibodies [6] and thus with serum from PCV2 infected pigs. Hence, some PCV2 vaccines rely on expressing the capsid protein and to form viral-like particles (VLPs), which activate the immunoresponse in pigs [7, 8]. Due to its strong immunogenicity, the capsid protein is the preferred antigen in a variety of serological tests.

Nowadays, PCV2 vaccines are available that generate an immune response against the viral capsid [9–11]. However, vaccination alone is not enough to control porcine circovirus-associated disease (PCVAD), because the antibody levels of piglets after administering the first vaccine dose may be low or even zero. Furthermore, one needs a specific protocol to determine the optimal time for early inoculation. Therefore, it is necessary to diagnose PCV2 in an efficient manner. Effective diagnosis ideally combines observing clinical signs, such as jaundice, weight loss, difficulty in breathing, enlarged lymph nodes, and diarrhea [12], as well as histopathology and serology tests. To speed up the process, both veterinarians and farmers need an accurate and rapid test to trace the disease to decide if the population at the farm is stable, or if they need to implement vaccination and biosecurity programs.

The most common methods for detecting PCV2 rely on bioassays: At present, PMWS is widely diagnosed via enzyme-linked immunosorbent assay (ELISA) targeting the antibody against PCV2, indirect immunofluorescence assay (IFA) [13, 14], and immunoperoxidase monolayer assay (IPMA) [8, 15]. Antibodies are widely accepted as the gold standard in terms of sensitivity and affinity, but they suffer from short shelf-life, high manufacturing costs, and relatively poor stability [16, 17].

Extensive efforts have been made to study the assembly of virus-like particles (VLP) and the specific PCV2-associated epitopes to provide a solid foundation for the development of engineered PCV2 vaccines [18]. Most of these vaccines consist of the ORF2 subunit antigen, which encodes the major capsid protein of PCV2. The carboxyl terminus of this capsid protein facilitates its self-assembly into capsid-like particles or VLPs [19], erratum at [20]. The expressed ORF2 gene product has a molecular mass of 30 kDa, similar to that found in purified virus particles [21]. Such VLPs can trigger immune responses in pigs, like native PCV2, and are extremely small, with a diameter of 17–22 nm [22]. The self-assembly of PCV2 capsid proteins forms an icosahedral capsid, making the virus highly stable in the environment and resistant to certain disinfectants. Thus, we used PCV2 VLPs as the template and analyte in this study instead of pathogenic viruses. Given the high similarity between the artificial VLP and the virus, this seems a valid approach.

Molecularly imprinted polymer nanoparticles (nano-MIPs) result from template-induced formation of specific recognition sites in a polymer [23]. In brief: functional and cross-linking monomers are polymerized in the presence of a template, often the intended analyte. Removing the template leaves behind recognition cavities in the polymer matrix which match it in terms of size, shape, and functionality. The resulting molecularly imprinted polymer (MIP) can bind the target selectively [24, 25]. Synthetic MIPs are an alternative to natural receptors that, for instance, have proven very useful as plastic antibodies in pseudo-immunoassays [26]. Recent studies report nano-MIPs for the detection of biomolecules such as human serum albumin [27], trypsin in human urine [28, 29], hepatitis A virus [30, 31], doxorubicin, and target toward epitope of EGFR [32], among others. In contrast to antibodies, they do not require using animals to produce them.

Fluorescence spectroscopy is widely used when studying the structures and conformations of proteins [33], especially when they interact with small molecules or nanoparticles [34]. The emission characteristics of tryptophan, tyrosine, and phenylalanine residues provide useful information for investigating binding mechanisms [35]. The quartz crystal microbalance (QCM) is a transducer that measures small mass changes on its surface. Its sensitivity has led to several studies on the binding of MIP to proteins [27, 36], and also viruses [37].

Herein, we report on nano-MIPs against PCV2 based on tertbutylacrylamide (TBAm), N-isopropylacrylamide (NIPAm), acrylic acid (AAC), N,N′-methylenebisacrylamide (BIS), and N-(3-aminopropyl) methacrylamide hydrochloride (APMA) and their use in assays based on QCM.

## Materials and methods

### Materials

PCV2 vaccine (Porcilis® PCV2), classical swine fever virus (CSFV) (or hog cholera) vaccine, and porcine reproductive and respiratory syndrome virus (PRRSV) vaccine (IngelvacPRRS® MLV) were provided by BF feeds Co. Ltd., Thailand. N-Tertbutylacrylamide (TBAm), N-isopropylacrylamide (NIPAm), acrylic acid (AAC), N,N′-methylenebisacrylamide (BIS), and N-(3-aminopropyl) methacrylamide hydrochloride (APMA) were the monomers and cross-linker in polymerization, respectively. Ammonium persulfate (APS) and N,N,N′,N′-tetramethylethylenediamine (TMED) were used as initiators for polymerization. 1-Ethyl-3-(3-dimethylaminopropyl) carbodiimide (EDC) and N-hydroxysuccinimide (NHS) served for protein coupling to cysteamine immobilized on the gold electrodes of QCM. Ethanol, toluene, acetone, and Milli-Q water were the solvents applied in the study. All chemicals were purchased from Sigma-Aldrich except milli-Q water, which was produced in-house.

### Nano-MIP synthesis

#### Solid-phase functionalization

**Fig. 1:**
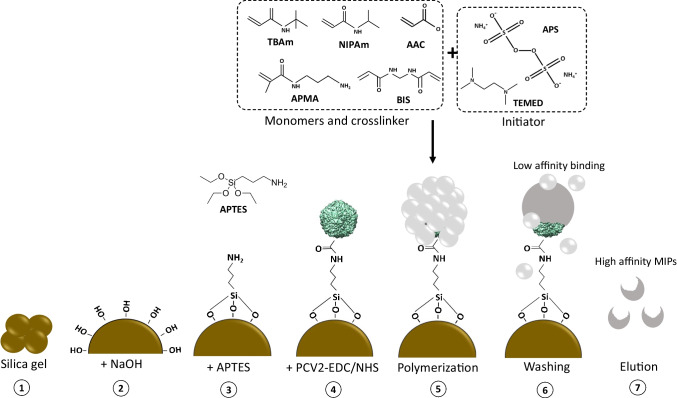
Schematic of synthesis PCV2 MIP nanoparticles against PCV2

Figure 1 sketches the synthesis scheme of nano-MIPs against PCV2 slightly amending a protocol published by the group of S. Piletsky [38]. First, we activated 2 g silica gel with particle size 60–200 µm by boiling it for 30 min in 5 ml 1 M aqueous NaOH solution to generate hydroxyl groups (-OH) on the surface. It was then washed with distilled water to neutral pH (approximately 7), filtrated, rinsed with acetone, and dried under vacuum in a Whatman filter no. 1. These matrices served as the support material for solid-phase synthesis. They were then silanized with 3-aminopropyltriethoxysilane (APTES) for introducing primary amino groups on the surface by incubating the activated silica gel in 4 ml APTES in toluene (2% (v/v)) in a tube rotator at room temperature overnight. The silica gels were then washed with acetone/ethanol (4:1) followed by excess acetone through glass Buechner filter funnel equipped with Whatman filter paper no.1 (porosity 100–160 µm) to dry them.

**Fig. 2:**
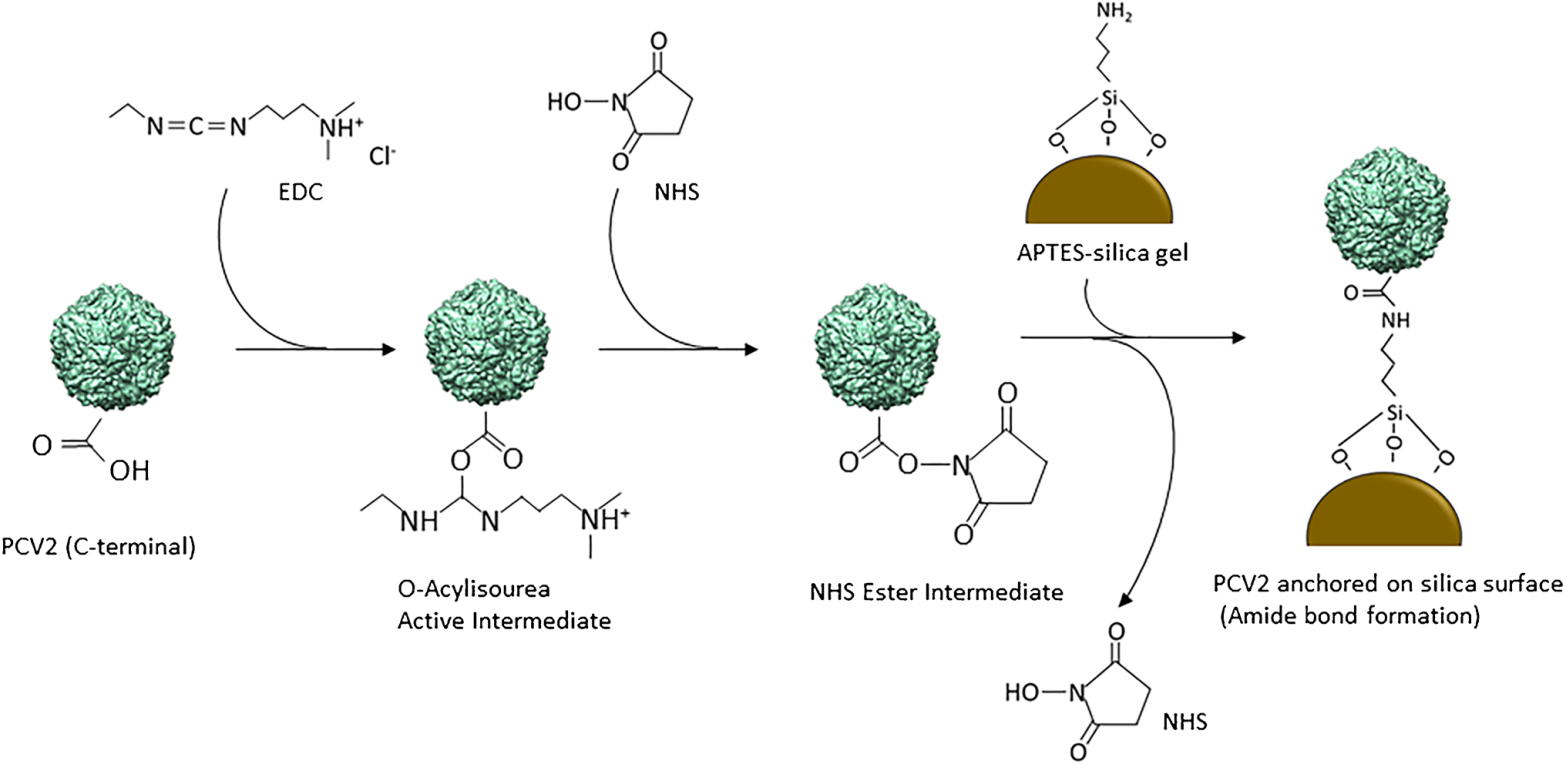
Conjugation reaction between C-terminus of PCV2 and amine-attached silica gel

Figure 2 shows the conjugation reaction between C-terminus of PCV2 and amine-attached silica gel: the first step involved activating the carboxylic group on PCV2. For that purpose, 5 ml of PCV2 (1:1000) was incubated with the coupling solution (at working concentration of 0.05 M of EDC and 0.1 M of NHS) for 15 min at room temperature. The activated PCV2 was mixed with activated silica gel from “Solid-phase functionalization” section and incubated at 4 °C overnight. To remove excess PCV2, silica gels were then washed with milli-Q water through a glass Buechner filter funnel as before. The final PCV2-modified silica gels were stored at 4 °C until use.

#### Nanoparticle synthesis

The monomer mix was prepared by dissolving 43 mg TBAm in 500 µl 95% ethanol and mixing with 50 mg of NIPAm, 4.6 mg BIS, 13.2 µL AAC, and 17.6 mg APMA. Afterwards, we adjusted with milli-Q water to a total volume of 4 mL. This solution was then sonicated for 10 min to complete dissolving. To initiate polymerization, 50 µl of 20% (w/v) APS and 50 µl of 20% (v/v) TMED were added to the mixture. The solutions containing monomers were immediately degassed by flushing with argon for 10 min and mixing with PCV2-derivatized silica gel, followed by degassing for 10 min. Polymerization took place at room temperature for 3 h. For non-imprinted polymer nanoparticles (nano-NIPs) used as a reference, the synthesis followed the same procedure, using the solid phase without PCV2.

#### Nanoparticle extraction

After synthesis, we first washed the silica gel with 100 mL milli-Q water (10 °C) through a Whatman filter no. 1 in a Buechner funnel to remove low-affinity nanoparticles and unreacted chemicals. To collect the high-affinity nano-MIPs, the silica gels were subsequently transferred into a syringe filter. Then, we added 10 mL milli-Q water (65 °C) into the syringe and incubated it in the water bath at 65 °C for 5 min to elute nano-MIPs. The elution step was repeated 4 times to collect nano-MIP solutions until the volume reached about 40 mL. The concentrations of nano-MIPs and nano-NIPs were calculated as mg/mL based on weighing the solid and as particle concentrations by considering the particle diameters.

### SEM characterization

Morphologies and sizes of nanoparticles were examined by scanning electron microscopy (SEM; Zeiss Supra 55VP). The dry silica gels after each step of the synthesis process were characterized. For both nano-MIPs and nano-NIPs, 10 µL of the respective particle solution with approximately 100 ppm concentration was dropped on a silicon wafer and dried at 80 °C for 10 min in an oven. For PCV2, CSFV, and PRRSV from vaccine, 10 µL of the diluted 1:2000 vaccine in milli-Q water was dropped on silicon wafer similar to the nanoparticles.

### Affinity binding analysis toward PCV2

Fluorescence spectrometry allowed us to evaluate binding affinities of both nano-MIPs and nano-NIPs toward PCV2 and selected viruses, respectively, through their quenching on a PerkinElmer LS 50B. For that purpose, we adjusted the reaction mixture with milli-Q water to a total volume of 2 mL after mixing 500 µL of PCV2 (1000 times dilution) with the corresponding nanoparticle solution (0, 25, 50, 75, 100, 150, 200, and 300 µg/mL). The reaction mixtures were incubated at room temperature for 1 min and then scanned between 280 and 380 nm (excitation = 270 nm, ex. slit = 4, scan speed = 50 nm/min). Fluorescence quenching data were then further analyzed using the Stern–Volmer equation [39] to assess affinity, as follows:

$$\frac{{F}_{0}}{F}=1+{k}_{\text{q}}{\tau }_{0}\left[Q\right]=1+{K}_{\text{SV}}\left[Q\right]$$

where *F*_0_ and *F* are the fluorescence intensity of PCV2 solution in the absence and presence of the quenching agent (nano-MIPs or nano-NIPs), respectively; *k*_q_ is the bimolecular quenching rate constant; *K*_SV_ is the Stern–Volmer quenching constant; *τ*_*0*_ is the average lifetime of the biomolecule without the quencher (a representative value is 10^−18^ s), and [*Q*] is the quencher concentration.

Moreover, the number of binding sites per particle and the binding constant of the interaction of nano-MIPs with PCV2 can be analyzed using the following equation [36].

$$\text{log}\frac{\Delta F}{F}=n\text{ log }\left[\text{Q}\right]+\text{log}{K}_{\text{b}}$$

where *ΔF* = *F*_0_ − *F*, *F*_0_ and *F* are the fluorescence intensities of PCV2 in the absence and presence of quencher, respectively, *n* is the number of binding sites, and *K*_b_ is the equilibrium binding constant.

### QCM measurements

#### Preparation of QCM electrode

**Fig. 3:**
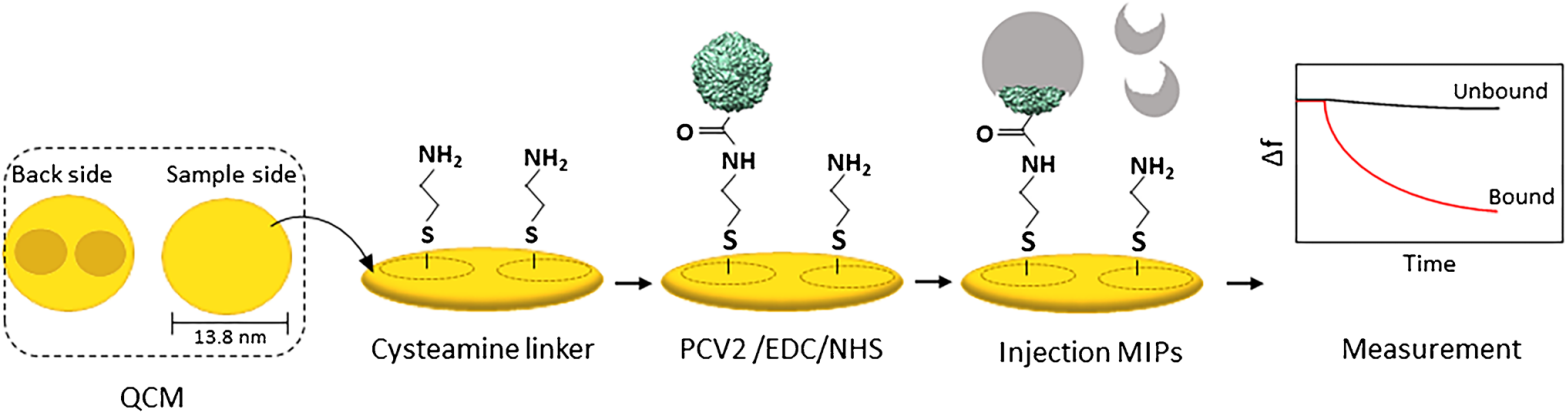
QCM modification and measurement of MIPs binding PCV2

We prepared QCM sensors by completely coating one side of AT-cut quartz plates (10 MHz, 13.8 mm diameter, purchased from Roditi Inc., UK) with 10% brilliant gold paste and thus fully metalizing it. The opposite side of the device was screen-printed with gold paste and burned at 400 °C for 4 h. Then, the sample side of the electrode was further coated and burned at 400 °C for 4 h (Fig. 3). QCM were characterized with a network analyzer (Agilent Technologies E5062A ENA series) to measure the respective damping spectra around the resonance frequency.

#### Modification steps

QCM electrodes were cleaned with acetone for 10 min and dried with nitrogen. To attach the linker to the electrode surface, 200 µL of 2.5 mM cysteamine solution was immobilized onto the electrode on the sample side and incubated in the dark at room temperature overnight. Then, the electrode was washed with milli-Q water and dried by blowing nitrogen over it. About 10 µL of PCV2 coupling solution (1:1000 PCV2 in 0.05 M EDC and 0.1 M NHS, incubated at 4 °C for 1 h) (Fig. 3) was dropped onto the electrode and incubated at 4 °C for 1 h to bind to cysteamine. Finally, we washed off excess PCV2 molecules with milli-Q water while the reference electrode contained only cysteamine. CSFV and PRRV for selectivity tests were immobilized using the same procedure as for PCV2.

#### QCM measurements

For sensor measurements, we inserted the respective QCM into a custom-made PDMS cell (sample volume ~ 150 µl) and connected it to an oscillator circuit monitored by frequency counter (Agilent HP5313A). Before measuring, the sensor was first flushed with milli-Q water to acquire a straight baseline. Subsequently, 200 µL suspension of nano-MIPs in milli-Q water was loaded into the measuring cell followed by waiting for a stable signal. To assess reproducibility, the same concentration of nano-MIPs was loaded 3 rounds in a row followed by washing, respectively. To record the sensor characteristics, we used various concentrations of nano-MIPs (25, 50, 100, and 200 mg/L) that were continuously loaded, i.e., without flushing with milli-Q water between individual exposures. For selectivity tests, we immobilized PCV2 on one of the two QCM electrodes and CSFV or PRRSV, respectively, on the other. Then, we measured after loading nano-MIPs and nano-NIPs, respectively.

Competitive assays relied on loading solutions containing mixtures of nano-MIPs with PCV2. In all cases, the corresponding frequency changes were read out into a computer by a custom-made LabView routine.

## Results and discussion

### Synthesis of nanoparticles and characterizing surface morphology

**Fig. 4:**
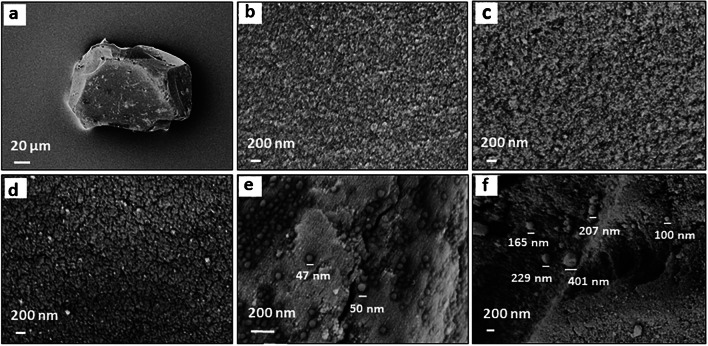
SEM images of **a** silica gel, and surface **b** before activation, **c** after activation with NaOH, **d** after treated with APTES attached with PCV2, **e** after polymerization and washing off low-affinity nanoparticles. In addition to the scale bars, **e** and **f **show lines that denote the diameters of selected particles

Figure 4 collects the SEM images of silica gel surfaces after each modification and immobilization step, respectively. As purchased, the silica gel consists of irregular particles having a diameter of about 150 µm (Fig. 4a) comprising a large number of pores on their surfaces (Fig. 4b). After activation, some pores crack, which leads to larger cavities (Fig. 4c). After treating it with 3-aminopropyltriethoxysilane (APTES), the silica surface appears rough and more granular (Fig. 4d). Surface modification with PCV2 and washing with milli-Q water clearly reveal PCV2 particles with a size of around 50 nm on the surface. After polymerization and washing the unbound and unreacted chemicals, nano-MIPs with various sizes from 100 to 400 nm are present on silica surfaces (Fig. 4f). This indicates that the synthesis of nano-MIPs has been successful. However, there are some PCV2 particles still attached to the silica surface without nano-MIPs having formed.

**Fig. 5:**
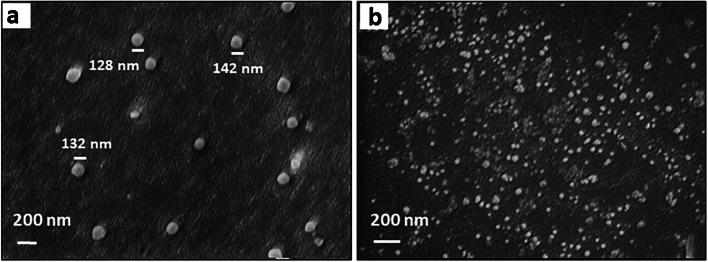
SEM images of **a** MIP nanoparticles and **b** NIP nanoparticles on silicon wafers

The nanoparticles were eluted with milli-Q water 65 °C and filtered through paper with a filter funnel to remove large particles. Figure 5a shows an SEM image revealing some of the resulting nano-MIPs: they are spherical with diameters around 120–150 nm. nano-NIPs are smaller with various sizes < 50 nm (see Fig. 5b). The comparably large difference in particle diameter may be the result of the fact that NIP particles do not only form on the surface, but also in solution, which means larger numbers, but smaller diameters. However, the details are still unclear and need further experiments.

### Characterization of virus particles

**Fig. 6:**
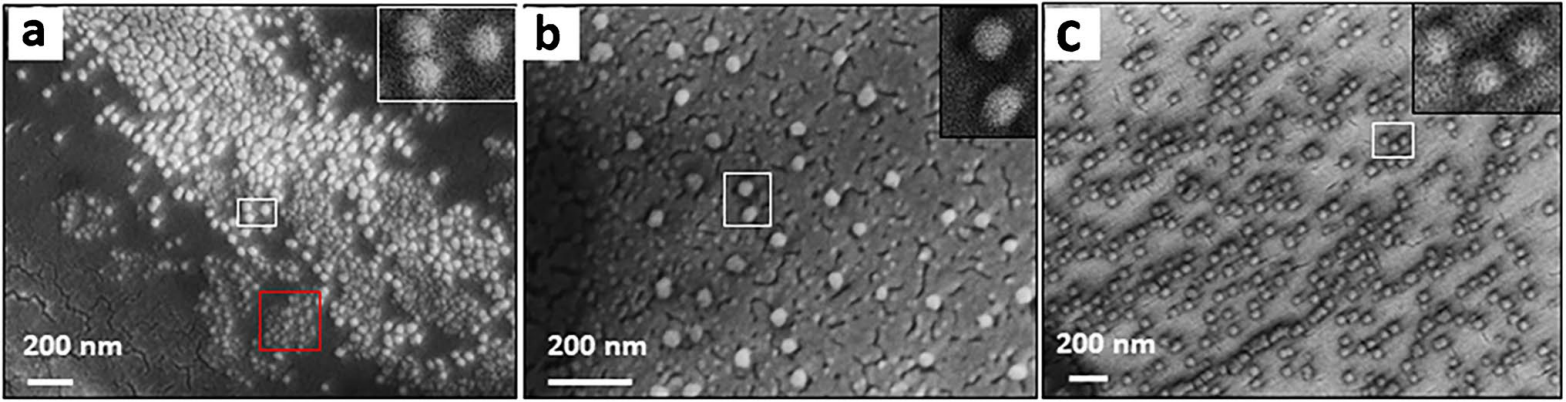
SEM images of virus particles from vaccine **a** PCV2, **b** CSFV, **c** PRRSV. The insets of each image show the areas marked with white boxes at higher magnification

Figure 6 shows SEM images of PCV2, CSFV, and PRRSV vaccine samples, respectively. For vaccine development, PCV2 capsid protein was assembled into regular homogenous viral-like particles (VLPs) with a diameter of 22 nm [40], which does not differ substantially from native PCV2 virion particles (*d* = 17 nm) [41]. PCV2 VLPs are spherical and have a diameter of 21 nm (red box, inset) and 50 nm (white box, inset) from expanding (Fig. 6a). This is in line with what one would expect: PCV2 vaccine relies on recombinant capsid to trigger the immune reactions of pigs. The classical swine fever vaccine is an attenuated live vaccine which is usually used to substitute CSFV (i.e., the pathogenic species) during selectivity test. Figure 6b shows CSFV virus particles with spherical shape and size approximately 50 nm similar to data in literature [42], while PRRSV vaccine, based on inactivated virus, was used as a substitute for PRRSV. The PRRSV particles are spherical with sizes around 45–70 nm (Fig. 6c), which matches a previous report [43]. This result indicates that PCV2 size is smaller than CSFV and PRRSV respectively.

### Fluorescence studies of the interaction between nanoparticles and PCV2

**Fig. 7:**
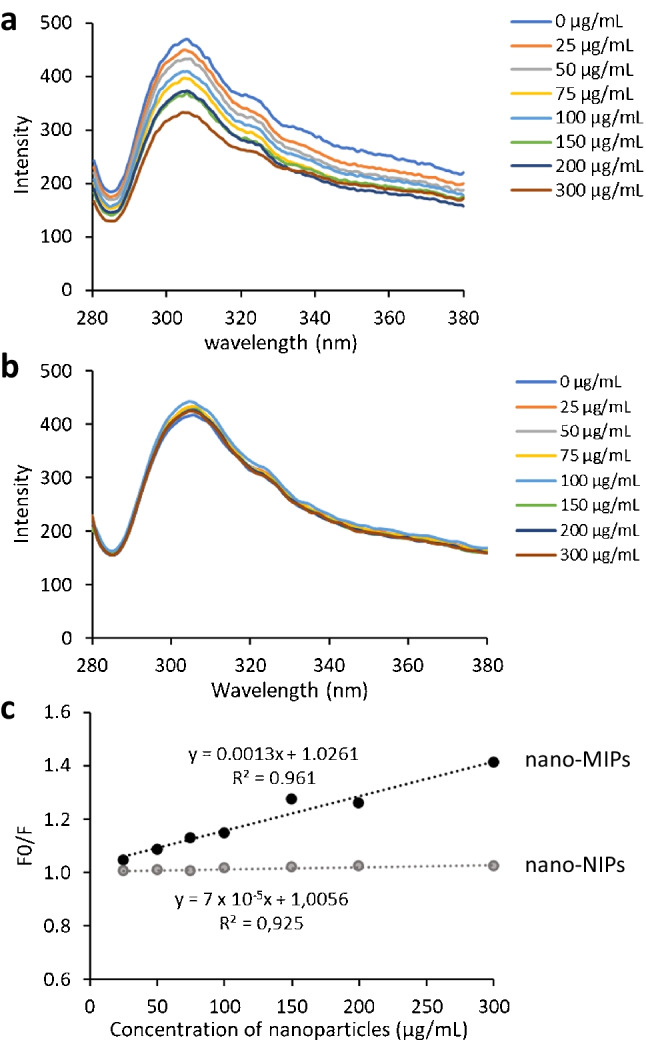
Fluorescence spectra of PCV2 in the presence of various concentrations of **a** nano-MIPs and **b** nano-NIPs. **c** Stern–Volmer plots for the binding of nano-MIPs and nano-NIPs nanoparticles to PCV2. The concentration of PCV2 was diluted 1:1000, and the concentrations of nanoparticles were 0–300 µg/mL

After adding nano-MIPs to solutions containing PCV2, they affect the emission intensity at 310 nm when excited at 275 nm as shown in Fig. 7a. Obviously, it decreases when increasing the nano-MIP concentration. The same procedure using nano-NIPs as a control does not cause any decrease in fluorescence of PCV2 (Fig. 7b). Figure 7c shows the Stern–Volmer plots for affinity interactions between nanoparticles and PCV2 with *F*_0_/*F* as a function of nano-MIP and nano-NIP concentrations, respectively (*F*_0_ is the fluorescence intensity of baseline, *F*_1_ is the fluorescence intensity of sample). The quenching rate constant for binding of nano-MIPs/PCV2 (*K*_SV_ = 1.3 × 10^−3^ µg/mL) is higher than for nano-NIPs/PCV2 (*K*_SV_ = 7 × 10^−5^ µg/mL). This clearly demonstrates that nano-MIPs bind to PCV2 with almost 18 times higher affinity than their non-imprinted counterparts. Further considering that the number concentrations of nano-NIPs are much higher than those of nano-MIPs (roughly by an order of magnitude), this even brings the difference in affinity higher than two orders of magnitude. From fluorescence intensity, the calculated limit of detection (LOD) of nano-MIPs was 47 µg/mL.

### QCM binding studies of PCV2 nano-MIPs

**Fig. 8:**
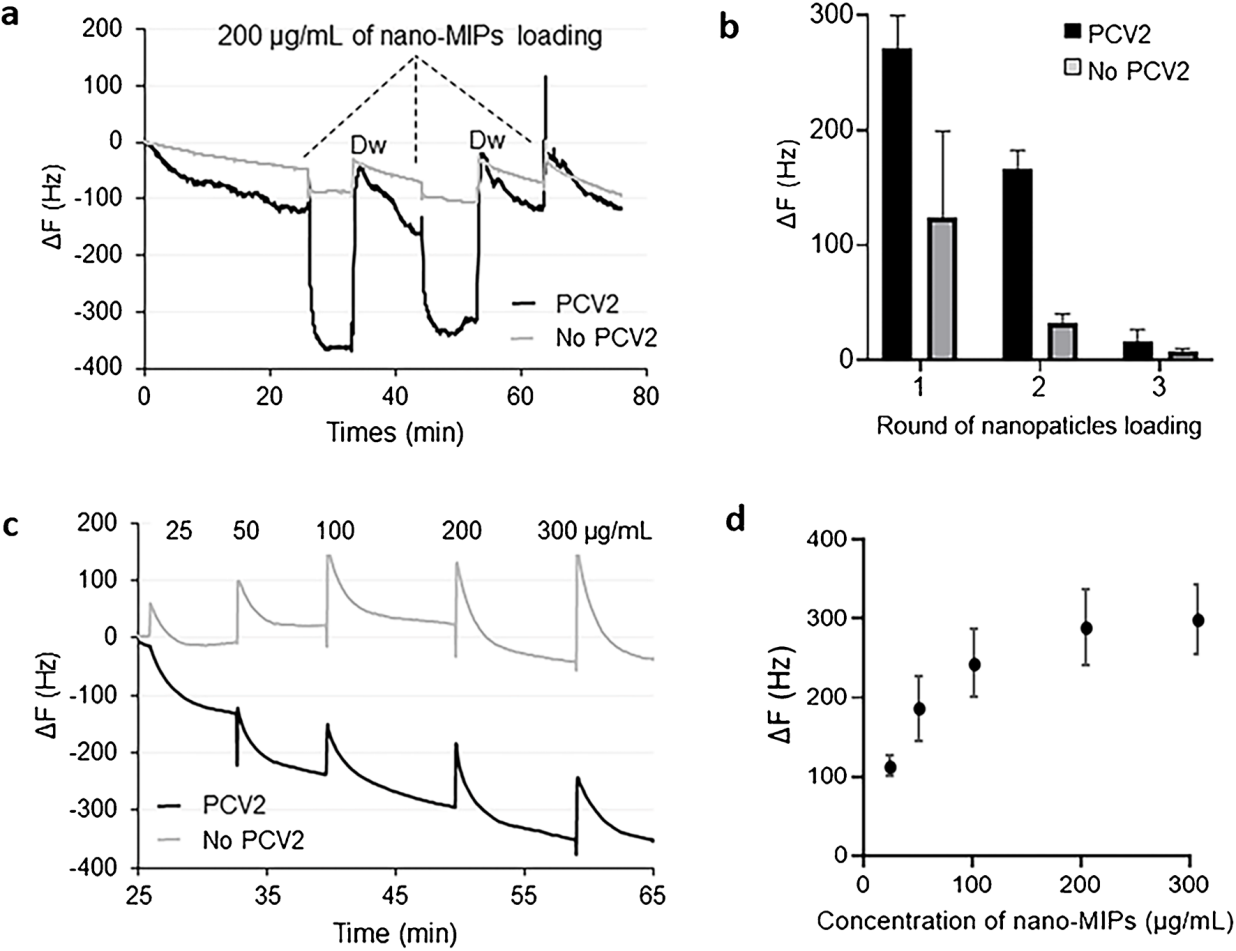
**a** QCM sensor responses for reproducibility test. **b** Reproducibility represented in bar graph. **c** Concentration-dependent QCM sensor responses for both PCV-coated channel (black line) and cysteamine-modified reference (gray line), and **d** corresponding sensor characteristic

In the first step, we tested sensitivity of the QCM assay (see Fig. 8). The measurements in this case use reversed logic as compared to “usual” direct nano-MIPs assays: the analyte — PCV2 — is immobilized on the surface of the QCM, which is exposed to samples containing the nano-MIPs. Most QCM assays work the other way round: they immobilize the receptor — the MIP in this case — and expose it to the analyte. To check reproducibility, the same concentration of nano-MIPs (200 µg/mL) was loaded three rounds in a row (see Fig. 8a, b). Channel 1 comprising PCV2 (black line) leads to five times higher signal responses than the reference channel (containing only the linker NH_2_, without PCV2) (red line), namely Δ*f* =  − 240 Hz and Δ*f* =  − 44 Hz, respectively. This clearly demonstrates that nano-MIPs indeed bind reversibly to the PCV2 particles immobilized on the QCM surface. Exposing the devices to nano-MIPs a second time results in somewhat smaller responses: channel 1 led to Δ*f* =  − 175 Hz and channel 2 to Δ*f* =  − 34 Hz. However, again the response of channel 1 is five times higher than that of channel 2. When injecting nanoparticles for the third time, there is only small signal response, which indicates saturation of the surface with nanoparticles. However, there is a seeming discrepancy between the results of fluorescence and QCM: whereas in the former case, the nano-MIPs show 18 times higher signals than the nano-NIPs, the latter only yield a factor of 5. The reason for this may be that the fluorescence assay takes place in solution, whereas QCM measurements use PCV2 immobilized on the device surface. It is valid to assume that they are thus sterically hindered compared to a suspension in a buffer, which may reduce selective binding.

Figure 8c shows the resulting sensor characteristic determined at nano-MIPs concentrations of 25, 50, 100, and 200 µg/mL leading to Δ*f* =  − 129, − 230, − 288, and − 348 Hz, respectively. At about 300 µg/mL, the sensor reaches saturation with Δ*f* =  − 354 Hz. For control, nano-NIPs showed negligible no interaction on channel 2, which contained only cysteamine molecules immobilized on the gold surface. The saturation frequency corresponds to roughly half a monolayer of polymer particles: using the Sauerbrey equation, a layer of nano-MIPs with 150-nm diameter should yield Δ*f* =  − 750 Hz. Interestingly, the NIP particles lead to slight non-Sauerbrey behavior, i.e., positive frequency shifts. Such behavior has been reported previously [44]. Figure 8d shows the resulting sensor characteristics. The limit of detection was calculated from the slope of the dynamic range (0–100 µg/mL) resulting in equally 35 µg/mL.

### Selectivity

**Fig. 9:**
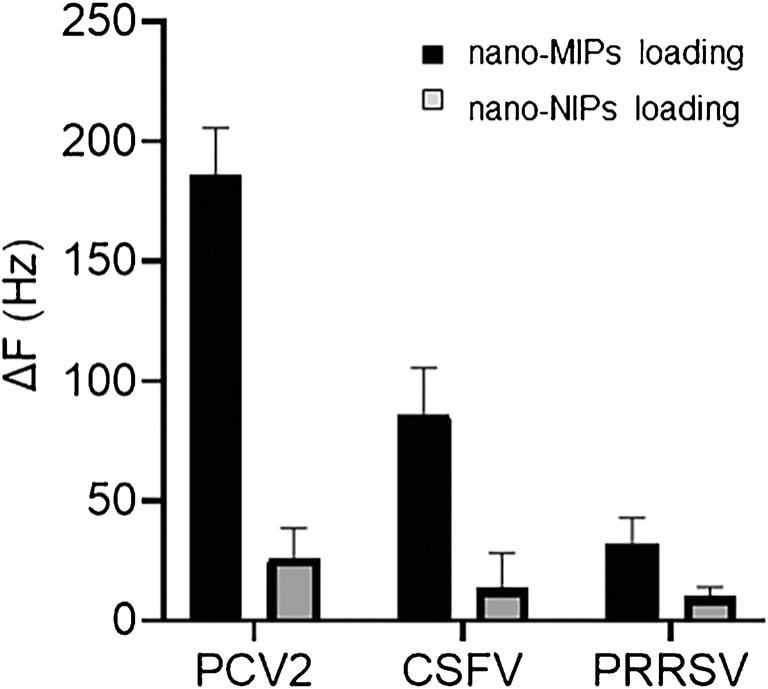
QCM selectivity patterns against PCV2, CSFV, and PRRSV. Black columns show nano-MIP data, gray ones nano-NIP

Selectivity tests relied on three different vaccines that differ from each other also in the way they are produced: PCV2 vaccine results from capsid protein expression; the unit of vaccine is presented in log2 ELISA units (ELISA absorbance values produced by twofold serial dilution). In contrast, CSFV and PRRSV are presented in TCID_50_ (means 50% tissue culture infective dose) [43, 45]. The respective solutions all contain the same virus concentrations (i.e., for each different type). We assessed the selectivity of nano-MIPs against the VLPs found in pig, e.g., CSFV and PRRSV. Selectivity tests relied on QCM containing immobilized PCV2 on one electrode and CSFV or PRRSV on the other electrode. Figure 9 shows the corresponding results: The signal response of the PCV2-coated channel is always higher than that of the respective competitor, i.e., CSFV or PRRSV. The frequency shifts caused by nano-MIPs are Δ*f* =  − 173 Hz (PCV2), − 100 Hz (CSFV), and − 40 Hz (PRSSV), respectively. At the same time, nano-NIPs (i.e., the non-imprinted reference; gray bar) hardly lead to any difference in binding affinity between the different viruses. Overall, nonspecific interactions between the polymers and the virus particles are almost negligible and equal for the three virus types. Hence, nano-MIPs show appreciable selectivity: even though they bound to PCV2 as well as to both CSFV and PRRSV, the selectivity factors are 2 and 5, respectively. Given that SEM images (Fig. 6) revealed that they are rather similar in size (50 nm), this is a very appreciable outcome, because it shows that recognition indeed relies on the surface chemistry of the respective viruses. For PRRSV, the selectivity factor is about a factor of 4 higher (20% instead of 5%) than for antibody-based sensing, which further corroborates the feasibility of the MIP approach [46].

### Competitive assay

**Fig. 10:**
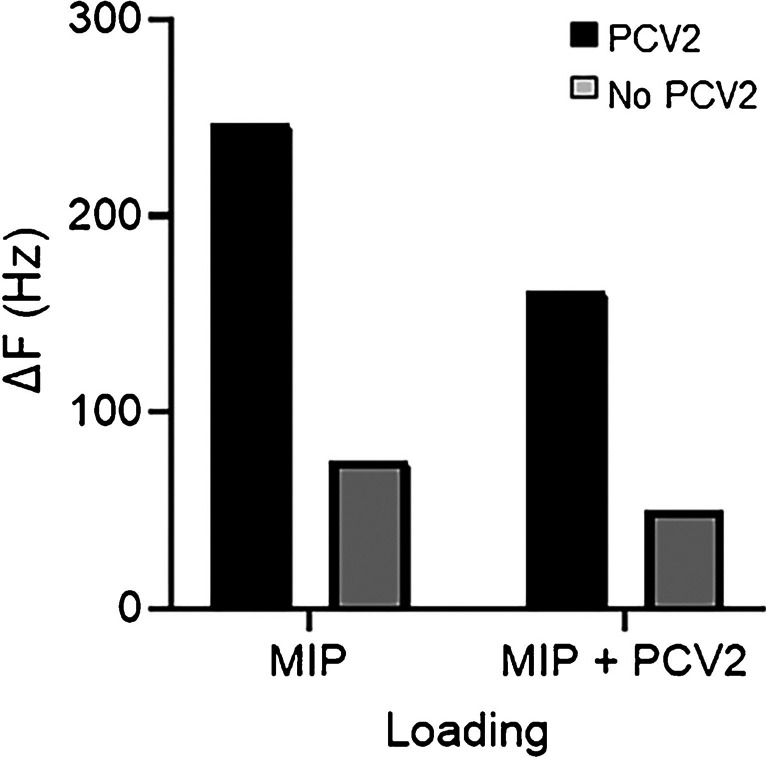
Competitive QCM measurements with PCV2 sensor

Nano-MIPs resulting from solid-phase synthesis claim that they contain only one binding site per particle [38], which makes them interesting for biomimetic assay formats. To assess this, we carried out a straightforward competitive assay by comparing the QCM sensor responses resulting from injecting 200 µg/mL of nano-MIPs and 200 µg/mL of nano-MIPs mixed with PCV2 (1000 times dilution). Figure 10 shows the corresponding sensor responses: when exposing the sensor surface to nano-MIPs without PCV2, one can observe Δ*f* =  − 274 Hz, whereas the mixture yields Δ*f* =  − 162 Hz, i.e., less. This clearly indicates that about 40% of the nano-MIPs in solution have already bound to PCV2 and thus no longer interact with the protein molecules immobilized on the sensor surface.

## Conclusion

Data from both fluorescence quenching and QCM clearly indicate binding between the synthesized nano-MIP and their target, PCV2 capsid protein. It is safe to assume that this takes place through different non-covalent interactions [47]. The imprinting effect observed depends on whether PCV2 is immobilized on a sensor surface, or not, which also indicates non-covalent binding at specific areas of the protein. Overall, this proof-of-concept work demonstrates that it is in principle possible to use artificial MIP nanobodies instead of (monoclonal) antibodies in detecting this veterinary disease: they bind to the virus capsid in a concentration-dependent manner. Also, the approach yields appreciable selectivity factors toward CSFV and PRRSV which have similar diameter as PCV2. Thus, they are inherently suitable to replace biological antibodies on rapid, low-cost assay formats.

## References

[1] Seeliger FA, Brugmann ML, Kruger L, Greiser-Wilke I, Verspohl J, Segales J, et al. Porcine circovirus type 2-associated cerebellar vasculitis in postweaning multisystemic wasting syndrome (PMWS)-affected pigs. Vet Pathol. 2007;44(5):621–34. 10.1354/vp.44-5-621. 17846234 10.1354/vp.44-5-621

[2] Park C, Seo HW, Han K, Chae C. Comparison of four commercial one-dose porcine circovirus type 2 (PCV2) vaccines administered to pigs challenged with PCV2 and porcine reproductive and respiratory syndrome virus at 17 weeks postvaccination to control porcine respiratory disease complex under Korean field conditions. Clin Vaccine Immunol. 2014;21(3):399–406. 10.1128/CVI.00768-13. 24403524 10.1128/CVI.00768-13 PMC3957680

[3] Choi J, Stevenson GW, Kiupel M, Harrach B, Anothayanontha L, Kanitz CL, et al. Sequence analysis of old and new strains of porcine circovirus associated with congenital tremors in pigs and their comparison with strains involved with postweaning multisystemic wasting syndrome. Can J Vet Res. 2002;66(4):217–24. 12418776 PMC227008

[4] Meehan BM, McNeilly F, Todd D, Kennedy S, Jewhurst VA, Ellis JA, et al. Characterization of novel circovirus DNAs associated with wasting syndromes in pigs. J Gen Virol. 1998;79(Pt 9):2171–9. 10.1099/0022-1317-79-9-2171. 9747726 10.1099/0022-1317-79-9-2171

[5] Huang L, Van Renne N, Liu C, Nauwynck HJ. A sequence of basic residues in the porcine circovirus type 2 capsid protein is crucial for its co-expression and co-localization with the replication protein. J Gen Virol. 2015;96(12):3566–76. 10.1099/jgv.0.000302. 26415571 10.1099/jgv.0.000302

[6] Mahe D, Blanchard P, Truong C, Arnauld C, Le Cann P, Cariolet R, et al. Differential recognition of ORF2 protein from type 1 and type 2 porcine circoviruses and identification of immunorelevant epitopes. J Gen Virol. 2000;81(Pt 7):1815–24. 10.1099/0022-1317-81-7-1815. 10859388 10.1099/0022-1317-81-7-1815

[7] Yang D, Chen L, Duan J, Yu Y, Zhou J, Lu H. Investigation of Kluyveromyces marxianus as a novel host for large-scale production of porcine parvovirus virus-like particles. Microb Cell Fact. 2021;20(1):24. 10.1186/s12934-021-01514-5. 33494762 10.1186/s12934-021-01514-5 PMC7836160

[8] Khayat R, Wen K, Alimova A, Gavrilov B, Katz A, Galarza JM, et al. Structural characterization of the PCV2d virus-like particle at 3.3 Å resolution reveals differences to PCV2a and PCV2b capsids, a tetranucleotide, and an N-terminus near the icosahedral 3-fold axes. Virology. 2019;537:186–97. 10.1016/j.virol.2019.09.001. 31505320 10.1016/j.virol.2019.09.001 PMC6958667

[9] Karuppannan AK, Opriessnig T. Porcine circovirus type 2 (PCV2) vaccines in the context of current molecular epidemiology. Viruses. 2017;9(5):99. 28481275 10.3390/v9050099 PMC5454412

[10] Wang L, Zhao D, Sun B, Yu M, Wang Y, Ru Y, et al. Oral vaccination with the porcine circovirus type 2 (PCV-2) capsid protein expressed by Lactococcus lactis induces a specific immune response against PCV-2 in mice. J Appl Microbiol. 2020;128(1):74–87. 31574195 10.1111/jam.14473

[11] Opriessnig T, Xiao C-T, Halbur PG, Gerber PF, Matzinger SR, Meng X-J. A commercial porcine circovirus (PCV) type 2a-based vaccine reduces PCV2d viremia and shedding and prevents PCV2d transmission to naive pigs under experimental conditions. Vaccine. 2017;35(2):248–54. 27919634 10.1016/j.vaccine.2016.11.085 PMC5221148

[12] Segalés J. Porcine circovirus type 2 (PCV2) infections: clinical signs, pathology and laboratory diagnosis. Virus Res. 2012;164(1–2):10–9. 10.1016/j.virusres.2011.10.007. 22056845 10.1016/j.virusres.2011.10.007

[13] Nawagitgul P, Harms PA, Morozov I, Thacker BJ, Sorden SD, Lekcharoensuk C, et al. Modified indirect porcine circovirus (PCV) type 2-based and recombinant capsid protein (ORF2)-based enzyme-linked immunosorbent assays for detection of antibodies to PCV. Clin Diagn Lab Immunol. 2002;9(1):33–40. 11777826 10.1128/CDLI.9.1.33-40.2002 PMC119869

[14] Lyoo KS, Joo HS, Davies PR, Han JH. Comparison of Porcine circovirus type 2 (PCV2) infection in light and heavy pigs of market age on farms with routine PCV2 vaccination. Can J Vet Res. 2012;76(3):221–3. 23277702 PMC3384286

[15] Farnham MW, Choi YK, Goyal SM, Joo HS. Isolation and characterization of porcine circovirus type-2 from sera of stillborn fetuses. Can J Vet Res. 2003;67(2):108–13. 12760475 PMC227037

[16] Lu R-M, Hwang Y-C, Liu IJ, Lee C-C, Tsai H-Z, Li H-J, et al. Development of therapeutic antibodies for the treatment of diseases. J Biomed Sci. 2020;27(1):1. 10.1186/s12929-019-0592-z. 31894001 10.1186/s12929-019-0592-z PMC6939334

[17] Parray HA, Shukla S, Samal S, Shrivastava T, Ahmed S, Sharma C, et al. Hybridoma technology a versatile method for isolation of monoclonal antibodies, its applicability across species, limitations, advancement and future perspectives. Int Immunopharmacol. 2020;85:106639-. 10.1016/j.intimp.2020.106639. 32473573 10.1016/j.intimp.2020.106639 PMC7255167

[18] Mo XB, Li XD, Yin B, Deng JH, Tian KG, Yuan A. Structural roles of PCV2 capsid protein N-terminus in PCV2 particle assembly and identification of PCV2 type-specific neutralizing epitope. Plos Pathog. 2019;15(3). 10.1371/journal.ppat.1007562. 10.1371/journal.ppat.1007562 PMC6415871 30822338

[19] Zhan Y, Yu WT, Cai X, Lei XN, Lei HY, Wang AB, et al. The carboxyl terminus of the porcine circovirus type 2 capsid protein is critical to virus-like particle assembly, cell entry, and propagation. J Virol. 2020;94(9). 10.1128/jvi.00042-20. 10.1128/JVI.00042-20 PMC7163136 32075927

[20] Zhan Y, Yu WT, Cai X, Lei XN, Lei HY, Wang AB, et al. The carboxyl terminus of the porcine circovirus type 2 capsid protein is critical to virus-like particle assembly, cell entry, and propagation (vol 94, e00042–20, 2020). J Virol. 2021;95(9). 10.1128/jvi.02409-20. 10.1128/JVI.00042-20 PMC7163136 32075927

[21] Nawagitgul P, Morozov I, Bolin SR, Harms FA, Sorden SD, Paul PS. Open reading frame 2 of porcine circovirus type 2 encodes a major capsid protein. J Gen Virol. 2000;81:2281–7. 10.1099/0022-1317-81-9-2281. 10950986 10.1099/0022-1317-81-9-2281

[22] Luo QP, Zhou JQ, Tang WH, Jiang P, Wan X, Ahmed W, et al. Investigation and development of transient production process for porcine circovirus Type-2 (PCV2) capsid protein in HEK293F cells. Protein Expr Purif. 2023;208. 10.1016/j.pep.2023.106293. 10.1016/j.pep.2023.106293 37137401

[23] Mosbach K. Molecular imprinting. Trends Biochem Sci. 1994;19(1):9–14. 8140624 10.1016/0968-0004(94)90166-x

[24] Canfarotta F, Cecchini A, Piletsky S. CHAPTER 1 Nano-sized Molecularly Imprinted Polymers as Artificial Antibodies. Molecularly Imprinted Polymers for Analytical Chemistry Applications. The Royal Society of Chemistry; 2018. p. 1–27.

[25] Ahmad OS, Bedwell TS, Esen C, Garcia-Cruz A, Piletsky SA. Molecularly imprinted polymers in electrochemical and optical sensors. Trends Biotechnol. 2019;37(3):294–309. 10.1016/j.tibtech.2018.08.009. 30241923 10.1016/j.tibtech.2018.08.009

[26] Wackerlig J, Lieberzeit P. Molecularly imprinted polymer nanoparticles in chemical sensing@ Synthesis, characterisation and application. Sensors Actuators B Chem. 2015;207:144–57.

[27] Sudjarwo WAA, Dobler MT, Lieberzeit PA. QCM-based assay designs for human serum albumin. Anal Bioanal Chem. 2022;414(1):731–41. 10.1007/s00216-021-03771-0. 34950982 10.1007/s00216-021-03771-0 PMC8748353

[28] Xu J, Prost E, Haupt K, Tse Sum Bui B. Direct and sensitive determination of trypsin in human urine using a water-soluble signaling fluorescent molecularly imprinted polymer nanoprobe. Sensors Actuators B Chem. 2018;258:10–7. 10.1016/j.snb.2017.11.077.

[29] Xu J, Zhang Y, Williams JV. Development and optimization of a direct plaque assay for trypsin-dependent human metapneumovirus strains. J Virol Methods. 2018;259:1–9. 10.1016/j.jviromet.2018.05.012. 29807042 10.1016/j.jviromet.2018.05.012 PMC6089538

[30] Yang B, Gong H, Chen C, Chen X, Cai C. A virus resonance light scattering sensor based on mussel-inspired molecularly imprinted polymers for high sensitive and high selective detection of Hepatitis A Virus. Biosens Bioelectron. 2017;87:679–85. 10.1016/j.bios.2016.08.087. 27631682 10.1016/j.bios.2016.08.087

[31] Zheng G, Lu Q, Wang F, Jin Q, Teng M, Zhang N, et al. Selection of affinity peptides for the purification potential of porcine circovirus type 2 (PCV2) Cap virus-like particles (VLPs). RSC Adv. 2017;7(62):38911–4.

[32] Canfarotta F, Lezina L, Guerreiro A, Czulak J, Petukhov A, Daks A, et al. Specific drug delivery to cancer cells with double-imprinted nanoparticles against epidermal growth factor receptor. Nano Lett. 2018;18(8):4641–6. 10.1021/acs.nanolett.7b03206. 29969563 10.1021/acs.nanolett.7b03206

[33] Wang Q, Zhang Y, Wang X, Wu Y, Dong C, Shuang S. Dual role of BSA for synthesis of MnO2 nanoparticles and their mediated fluorescent turn-on probe for glutathione determination and cancer cell recognition. Analyst. 2019;144(6):1988–94. 10.1039/C8AN02501K. 30698591 10.1039/c8an02501k

[34] Baral A, Satish L, Das DP, Sahoo H, Ghosh MK. Construing the interactions between MnO2 nanoparticle and bovine serum albumin: insight into the structure and stability of a protein–nanoparticle complex. New J Chem. 2017;41(16):8130–9. 10.1039/C7NJ01227F.

[35] Wang Q, Dou X, Chen X, Zhao Z, Wang S, Wang Y, et al. Reevaluating protein photoluminescence: remarkable visible luminescence upon concentration and insight into the emission mechanism. Angew Chem Int Ed Engl. 2019;58(36):12667–73. 10.1002/anie.201906226. 31243877 10.1002/anie.201906226

[36] Roy AS, Tripathy DR, Chatterjee A, Dasgupta S. A spectroscopic study of the interaction of the antioxidant naringin with bovine serum albumin. J Biophys Chem. 2010;01(03):12. 10.4236/jbpc.2010.13017.

[37] Klangprapan S, Choke-Arpornchai B, Lieberzeit PA, Choowongkomon K. Sensing the classical swine fever virus with molecularly imprinted polymer on quartz crystal microbalance. Heliyon. 2020;6(6):e04137-e. 10.1016/j.heliyon.2020.e04137. 32548329 10.1016/j.heliyon.2020.e04137 PMC7284075

[38] Canfarotta F, Poma A, Guerreiro A, Piletsky S. Solid-phase synthesis of molecularly imprinted nanoparticles. Nat Protoc. 2016;11(3):443–55. 10.1038/nprot.2016.030. 26866789 10.1038/nprot.2016.030

[39] Wang G, Liu X, Yan C, Bai G, Lu Y. Probing the binding of trypsin to glutathione-stabilized gold nanoparticles in aqueous solution. Colloids Surf B Biointerfaces. 2015;135:261–6. 10.1016/j.colsurfb.2015.07.063. 26263214 10.1016/j.colsurfb.2015.07.063

[40] Masuda A, Lee JM, Miyata T, Sato T, Hayashi S, Hino M, et al. Purification and characterization of immunogenic recombinant virus-like particles of porcine circovirus type 2 expressed in silkworm pupae. J Gen Virol. 2018;99(7):917–26. 10.1099/jgv.0.001087. 29851377 10.1099/jgv.0.001087

[41] Wang H, Zhang K, Lin C, Zhou J, Jin Y, Dong W, et al. Conformational changes and nuclear entry of porcine circovirus without disassembly. J Virol. 2019;93(20). 10.1128/jvi.00824-19. 10.1128/JVI.00824-19 PMC6798103 31341057

[42] Wang R, Zhi Y, Guo J, Li Q, Wang L, Yang J, Jin Q, Wang Y, Yang Y, Xing G, Qiao S, Zhao M, Deng R, Zhang G. Efficient purification of cell culture-derived classical swine fever virus by ultrafiltration and size-exclusion chromatography. Front Agr Sci Eng. 2015;2(3):230–6. 10.15302/j-fase-2015071.

[43] Liang Z, Li P, Wang C, Singh D, Zhang X. Visualizing the transport of porcine reproductive and respiratory syndrome virus in live cells by quantum dots-based single virus tracking. Virologica Sinica. 2020;35(4):407–16. 10.1007/s12250-019-00187-0. 31872331 10.1007/s12250-019-00187-0 PMC7462959

[44] Latif U, Can S, Hayden O, Grillberger P, Dickert FL. Sauerbrey and anti-Sauerbrey behavioral studies in QCM sensors—Detection of bioanalytes. Sens Actuators B Chem. 2013;176:825–30. 10.1016/j.snb.2012.09.064.

[45] Hao G, Zhang H, Chen H, Qian P, Li X. Comparison of the Pathogenicity of Classical Swine Fever Virus Subgenotype 2.1c and 2.1d strains from China. Pathogens. 2020;9(10):821. 33036431 10.3390/pathogens9100821 PMC7600237

[46] Luo BB, Wu SX, Zou WG, Zhang ZH, Zhao MF, Shi SH, et al. Label-free immunoassay for porcine circovirus type 2 based on excessively tilted fiber grating modified with staphylococcal protein A. Biosens Bioelectron. 2016;86:1054–60. 10.1016/j.bios.2016.07.100. 27518582 10.1016/j.bios.2016.07.100

[47] Chen B, Piletsky S, Turner AP. High molecular recognition: design of “Keys.” Comb Chem High Throughput Screen. 2002;5(6):409–27. 10.2174/1386207023330129. 12470272 10.2174/1386207023330129

